# No Changes in Keratometry Readings and Anterior Chamber Depth after XEN Gel Implantation in Patients with Glaucoma

**DOI:** 10.3390/jcm13092537

**Published:** 2024-04-26

**Authors:** Filippo Tatti, Claudia Tronci, Filippo Lixi, Giuseppe Demarinis, Sviatlana Kuzmich, Enrico Peiretti, Maurizio Fossarello, Giuseppe Giannaccare

**Affiliations:** Department of Surgical Sciences, Eye Clinic, University of Cagliari, 09124 Cagliari, Italy; filippo.tatti@unica.it (F.T.); c.tronci5@studenti.unica.it (C.T.); f.lixi1@studenti.unica.it (F.L.); demarinis91@gmail.com (G.D.); s.kuzmich@studenti.unica.it (S.K.); enripei@hotmail.com (E.P.); maurizio.fossarello@gmail.com (M.F.)

**Keywords:** XEN, glaucoma surgery, astigmatism, keratometry, anterior chamber depth

## Abstract

**Background:** This study aimed to compare keratometry and anterior chamber depth (ACD) changes after XEN implantation in primary open-angle glaucoma (POAG) cases over a 3-month follow-up period. **Methods:** Twenty patients with POAG who underwent XEN63 implantation, either standalone or combined with cataract surgery, were included. Preoperative data, including best-corrected visual acuity (BCVA), refraction, gonioscopy, ophthalmoscopy, intraocular pressure (IOP) evaluation, and axial length, were collected. Corneal topography and ACD measurements were assessed preoperatively and at postoperative days 1, 7, 15, 30, 60, and 90. Each patient’s eye that underwent XEN surgery was included in the study group, with the fellow eye serving as a control. **Results:** In the study group, there was a significant decrease in IOP after XEN stent implantation at all investigated time intervals (*p* < 0.05). However, changes in mean ACD did not show statistically significant differences at any follow-up examination in both the study and control groups. Additionally, keratometry readings revealed no significant changes in total astigmatism or steep keratometry values in either group. **Conclusions:** XEN implantation in POAG cases resulted in a significant decrease in IOP over the 3-month follow-up period. However, there were no significant changes observed in mean ACD or keratometry readings, indicating stability in these parameters post-XEN implantation. These findings suggest that XEN implantation may be an effective option for IOP reduction without affecting corneal curvature or ACD in POAG patients.

## 1. Introduction

Glaucoma is a diverse collection of chronic, progressive optic neuropathies. The estimated prevalence of this condition is 2.9% in Europe and 3.5% worldwide among those aged 40 years and older [1]. Currently, the evidence-based and widely accepted therapeutic approach for glaucoma patients is a decrease in intraocular pressure (IOP) [2].

If the medical treatment fails to halt the progression of glaucomatous damage or adversely impacts quality of life, an assessment of alternative approaches, such as selective laser trabeculoplasty (SLT) or surgical intervention, becomes imperative [2]. Numerous factors must be taken into account when determining the most effective management. These include compliance, the stage of glaucoma, and the likelihood of success with medical or laser treatment [2].

Despite being the gold standard surgical operation, trabeculectomy (TE) is characterized by an invasive nature, comparatively high-risk profile, and the potential for complications. Among them, the encapsulation of the filtering bleb is the most prevalent, occurring in 4% to 30% of cases; it causes an altered outflow and a gradual increase in IOP [3,4]. Additional common and worrisome complications include hypotonia and flat anterior chamber (FAC) [5]. Edmunds et al. determined that the incidence of hypotony and FAC was 23.9% and 24.3%, respectively [6]. If not appropriately managed, the aforementioned conditions may give rise to further complications, such as cataract development, synechiae, persistent choroidal detachment or hypotony maculopathy, and ultimately, prolonged or permanent visual impairment [7]. Furthermore, a partial, temporary reduction in visual acuity is often observed subsequent to trabeculectomy surgery, although the precise underlying factors contributing to this reduction have not been completely clarified. It is not only attributed to changes in blood pressure, FAC, hypotony, choroidal detachment, wipe-out syndrome, and complications related to local anaesthesia [8].

Some studies have suggested a possible correlation between changes in visual acuity following TE and refractive changes [9]. A significant incidence of astigmatism following TE surgery has been observed, exhibiting a mid-to-long persistence (10–12 months) [10,11]. A persistence of astigmatism was noted by some authors among patients who received subconjunctival MMC to reduce complications related to fibrosis [12].

Nevertheless, refractive changes could also be correlated with anterior chamber depth (ACD) modifications [13]. Indeed, during the early post-trabeculectomy period, variations in ACD may occur, and these changes could be related to fluctuations in IOP and modifications of bleb functionality [14].

The impact of minimally invasive glaucoma surgeries (MIGS) on refractive changes and ACD variations still represents an open issue that deserves further exploration. Various techniques are categorized under the acronym MIGS according to the space in which the aqueous humour is drained [15,16]. There is increasing clinical interest in the field of MIGS, specifically in newer approaches named ‘minimally invasive bleb surgery’ (MIBS). The latter combines the filtering properties of traditional glaucoma surgery with those of MIGS. Among these, the XEN Gel Implant (AbbVie Company, Irvine, CA, USA) is a porcine gelatine 6 mm-long tube designed to create a channel from the anterior chamber to the subconjunctival space. The stent comes preloaded in an injector and is usually inserted in the iridocorneal angle via an ab interno technique with a small corneal incision, following a subconjunctival injection of 0.1 mL mitomycin C (MMC) 0.02% [17,18].

Currently, the literature lacks sufficient information regarding the refractive effects of the XEN Gel Implant [8]. The purpose of this study is to investigate keratometry and ACD changes following XEN Gel implantation in a group of patients with glaucoma. Additionally, it seeks to establish a correlation between the ACD variations and the IOP value.

## 2. Materials and Methods

This study included 40 eyes of 20 glaucoma patients. For each patient, one eye underwent XEN63 implantation and the fellow eye acted as a control. The subjects were recruited from a prospective cohort conducted at a single centre (University Hospital of Cagliari, Cagliari, Italy). The patients underwent surgery between October 2022 and July 2023. The study received approval by the Institutional Review Board (IRB) of the University of Cagliari (PG/2022/341; 26 January 2022). All subjects provided written informed consent and the study was performed in accordance with the Declaration of Helsinki.

Preoperative data were collected for each patient, including best-corrected visual acuity (BCVA), refraction, examination of the anterior segment with a slit lamp, gonioscopy, ophthalmoscopy, IOP evaluation, and axial length. In addition, corneal topography and anterior chamber depth measurements were assessed using the Pentacam Comprehensive Eye Scanner (Oculus Optikgeraete GmbH, Wetzlar, Germany) and the OA-Optical Biometer TOMEY GmbH. These examinations were repeated at 1, 7, 15, 30, 60, and 90 days postoperatively. Additionally, both intraoperative and postoperative complications were gathered, as well. Numerical hypotony was regarded when the IOP was less than 6 mmHg. On the other hand, persistent hypotony was defined as an IOP less than 6 mmHg that was present at two consecutive postoperative visits that were more than 15 days apart.

### 2.1. Surgical Procedures

In all cases, XEN Gel implantation was performed under topical anesthesia by the same surgeon (F.T.). The operating field was prepared with adequate skin disinfection and sterile field dressing. In brief, an ab interno gel stent implantation (XEN63) was performed in the upper nasal quadrant following a subconjunctival injection of mitomycin C (0.1 mL, 0.02%). The AC was filled with a cohesive viscoelastic gel, the injector needle was inserted through a 1.8 mm infero-temporal corneal paracentesis incision, and directed towards the superonasal quadrant. The needle was then inserted into the sclera guided by the goniolens, slightly anterior to the nonpigmented trabecular meshwork, and the device was released after the needle appeared in the sub-conjunctival space posteriorly, approximately 2–3 mm from the limbus. Subsequently, the subconjunctival mobility of the implant was checked. Following hydration of the incisions, 0.1 ml of 1% cefuroxime was injected in the anterior chamber along with 4 mg/1 mL of dexamethasone phosphate subtenon (Decadron, Farmaceutici Caber SpA, Pomezia, Italy). Post-surgery care included a 2-week course of topical antibiotic for infection prophylaxis and a six-month regimen of gradually tapering topical corticosteroids.

### 2.2. Statistical Analysis

As previously reported by Senthil et al., to detect a 0.5-diopter (D) difference in astigmatism after surgery with a power of 80% and a type I error of 0.5, the required sample size was 20 eyes in each cohort [19]. For each patient, one eye underwent XEN surgery and the fellow eye acted as a control. Therefore, 40 eyes of 20 subjects with primary open-angle glaucoma (POAG) were enrolled in the study. Descriptive and exploratory data analyses were conducted. Statistical analysis was performed using IBM SPSS Statistics, version 25 for Windows. Means ± standard deviations (SDs) for continuous variables were estimated or percent distributions were presented. We examined the normality and the homogeneity of the continuous variables under investigation employing the Shapiro-Wilk test. Fisher’s test was used to compare categorical variables. Parametric (*t*-test) and non-parametric (Mann–Whitney test) tests were used to compare normally and non-normally distributed variables, respectively. Statistical analyses among groups were performed by Friedman’s test and a pairwise signed rank test. In addition, BCVA, astigmatism, and IOP changes were tested for possible correlation using Pearson’s test. Visual acuity was converted to logMAR for statistical analysis. A *p* value of <0.05 was considered statistically significant.

## 3. Results

### 3.1. Baseline Characteristics of the Study Population

In this study, 40 eyes of 20 Caucasian patients (12 males and 8 females) with primary open angle glaucoma (POAG) were included. The mean age was 64.3 years (range 44–83 years). The eye that underwent XEN implantation (study group, *n* = 20) and the fellow eye (control group, *n* = 20) were analyzed in each patient. In the study group, thirteen eyes (65%) were phakic and seven (35%) were pseudophakic. Eight eyes (40%) had a previous surgery. The control group included twelve phakic (60%) and eight pseudophakic eyes (40%). Eight eyes had a previous surgery (40%). In the study group, sixteen eyes underwent XEN implantation alone (80%) and four eyes underwent combined surgery with phacoemulsification (20%). Demographic data from the patients are summarized in Table 1.

### 3.2. Intraocular Pressure and Medications

Concerning eyes in the study group, after a follow-up period of 3 months, IOP exhibited a significant decrease from 28.75 ± 7.52 mmHg preoperatively to 14.30 ± 5.42 mmHg (*p* < 0.001), corresponding to a 50.26% reduction. The mean preoperative IOP dropped significantly after stent implantation at all investigated time intervals (*p* < 0.05).

In the control group, the mean preoperative IOP was 17.05 ± 3.68 mmHg, and the mean final IOP after 3 months was not significantly different, 15.65 ± 2.96 mmHg (*p* = 0.285). The mean number of preoperative medications was 2.65 ± 1.04 for the study group and 2.50 ± 0.83 for the control group. Postoperatively, the mean number of medications decreased significantly to 0.20 ± 0.52 (*p* < 0.001) in the study group and was not significantly different in the control group 2.65 ± 0.88 (*p* = 0.186).

### 3.3. BCVA Examination

In the study eyes, BCVA was 0.45 ± 0.55 logMAR at baseline and improved to 0.33 ± 0.57 logMAR at the final follow-up (*p* = 0.029), while in the control group, it was 0.10 ± 0.09 logMAR at baseline and 0.09 ± 0.09 logMAR (*p* = 0.6181) at the final follow-up.

### 3.4. Pentacam Parameter Evaluation

In the study group, the mean ACD was 3.37 ± 0.59 mm at baseline and 3.27 ± 0.55 mm, 3.30 ± 0.59 mm, and 3.32 ± 0.51 mm at 1 day, 1 month, and 3 months after surgery, respectively. In comparison, in the control group, the mean ACD was 3.44 ± 0.48 mm at baseline and 3.45 ± 0.46 mm, 3.41 ± 0.46 mm, and 3.38 ± 0.43 mm at 1 day, 1 month, and 3 months of follow up, respectively. In both groups, changes in the mean ACD did not reveal statistically significant differences at any of the follow-up examinations. When comparing the ACD results between the study and control groups, no statistically significant difference was detectable at baseline or at any follow-up visit (*p* > 0.05). Accordingly, in both groups, changes in ACD did not reveal statistically significant differences at any of the follow-up examinations. There was no statistically significant difference between the two groups during follow-up examinations (*p* > 0.05) (Table 2).

Moreover, even the total astigmatism and the steep did not change significantly in both groups. Specifically, in the study group, mean astigmatism was 0.94 ± 0.64 D at baseline and 0.91 ± 0.44 D, 0.89 ± 0.40 D, 0.93 ± 0.41 D, and 0.87 ± 0.39 D at 1 day, 7 days, 1 month, and 3 months after surgery, respectively. In control group, mean astigmatism was 0.80 ± 0.52 D at baseline and 0.77 ± 0.46 D, 0,73 ± 0.44 D, 0.75 ± 0.52, and 0.80 ± 0.56 D at 1 day, 7 days, 1 month, and 3 months of follow up. Similarly, the steep did not show significative changes in both groups [(in the study group, 99.3° ± 43.7° and 84.8° ± 31.9° at baseline and 3 months after surgery, respectively, *p* = 0.77) (in the control group, 97.1° ± 45.5° and 98.0° ± 40.1° at baseline and at 3 months of follow up, respectively, *p* = 0.92)] Further analysis showed no statistically significant intergroup differences at each follow-up visit (*p* > 0.05) (Table 2).

Furthermore, BCVA and astigmatism changes in the two groups were tested for a possible correlation. No significant results at baseline and at 1 month or 3 months of follow up were observed in the study group. Conversely, in the control group, a significant negative correlation was found at the 3-month follow-up (study group baseline: r = 0.15, *p* = 0.53; 1 month: r = −0. 13, *p* = 0.58; 3 months: r = 0.05, *p* = 0.83; and control group baseline: r = −0.37, *p* = 0.10; 1 month: r = −0.32, *p* = 0.17; 3 months: r = −0.47, *p* = 0.037). Moreover, a possible correlation between ACD and IOP variations was examined and a significant negative correlation was detected in the study group at 1 and 3 months after surgery. For the control group, no significant correlation was observed between IOP and ACD at each follow-up visit (study group baseline: r = 0.21, *p* = 0.36; 1 month: r = −0.49, *p* = 0.027; 3 months: r = −0.58, *p* = 0.007; and control group baseline: r = −0.06, *p* = 0.79; 1 month: r = −0.01, *p* = 0.96; 3 months: r = −0.02, *p* = 0.92).

### 3.5. Complications and Reoperations

There were no reports of intraoperative complications in the study group. During follow-up period, 11 eyes (55%) did not experience any postoperative complications. The most frequent complication in the remaining eyes (45%, *n* = 9) was numerical hypotony, with 88.89% of these cases resolving at week 2 without intervention. Persistent hypotony occurred in one eye (5%).

Two patients (10%) experienced a proximal ostium obstruction of the XEN, which was successfully treated with a YAG laser. Due to bleb flattening, filtration area fibrosis or distal XEN obstruction, seven eyes (35%) developed a decrease in bleb function, resulting in an IOP elevation. In all of these patients, a needling procedure with MMC injection was performed. The time to needling procedure ranged between 15 and 60 days (mean 30.85 ± 14.15) after XEN implantation

### 3.6. Surgical Outcomes

In the study group, complete success was achieved in 10 eyes (50%), and overall success in 17 eyes (85%). Qualified success was achieved in seven eyes (35%).

## 4. Discussion

MIGS has been shown to be both safe and effective at lowering IOP, as well as the number of glaucoma medications, in patients with mild to moderate disease [20]. The findings of our study are consistent with those that have been reported by various authors in the past, highlighting XEN as a valuable tool for reducing and controlling IOP in glaucoma patients [20]. Specifically, a statistically significant drop in IOP of 50.26% was observed during the 3-month follow-up, surpassing or mirroring outcomes from previously published studies. Indeed, various authors have documented the mid- and long-term efficacy of the XEN45 implant, both in terms of lowering IOP and reducing ocular hypotensive medications [21,22,23]. This holds true whether it is used alone or in conjunction with phacoemulsification surgery, particularly in POAG patients [24].

This procedure demonstrated safety in terms of complications that occurred during and after the operation, which is in line with the findings of other research [25]. The study group did not report any intraoperative complications. Only two patients (10%) encountered XEN device obstruction, which was effectively treated with YAG laser and needling procedures, as previously described by our group [26]. The second and most common complication was numerical hypotony, which was seen in 45% of the cases (*n* = 9). It was resolved in almost all of the cases (88.89%) by the second week without further intervention. Persistent hypotony occurred in one eye, and these transient and self-limited findings are comparable to records reported by other authors [25].

Our research additionally highlighted a significant negative correlation between IOP and ACD within the study group at both 1 and 3 months post-surgery. Similar findings were reported in other glaucoma procedures. Francis et al. observed a variable reduction in axial length, contingent on the degree of IOP reduction and the final postoperative IOP. They highlighted the importance of considering this alteration in axial length when anticipating the intended refractive outcome [27]. Nevertheless, in our study, despite a significant alteration in ACD, no significant changes in BCVA occurred.

In this context, an unexpected lack of significant change in keratometry was observed in both the study group and the control group. This finding contrasts with reports from various authors who documented significant changes in keratometry following various glaucoma procedures. Hugkulstone was the first author to investigate corneal astigmatism after trabeculectomy, describing a steepening of the vertical meridian [28]. However, no correlation was found between the decrease in IOP and changes in BCVA and corneal radii [28]. Other authors have also confirmed alterations in the keratometry index following trabeculectomy [11,12]. Additionally, researchers have studied this parameter in relation to various glaucoma devices. Hammel et al. reported only a transient modification of corneal astigmatism in 19 patients who underwent Ex-PRESS Miniature Glaucoma Implant surgery (Alcon Inc., Geneva, Switzerland), which was correlated with fluctuations in IOP and ACD [29]. More recently, Tzu et al. investigated refractive outcomes after glaucoma drainage device surgery, finding a mean induced astigmatism [30]. However, this study did not differentiate between the trabeculectomy and combined glaucoma drainage device populations [30]. A recent study compared surgically induced astigmatism following trabeculectomy and XEN implantation [9]. The authors showed a significant and comparable surgically induced astigmatism 3 months after surgery, which remained stable during the further follow-up period of 24 months. However, in contrast with this latter study that only analyzed objective and subjective refraction, our data did not show any significant changes in topographical analysis following XEN implantation.

The etiology of induced astigmatism following glaucoma procedures remains incompletely elucidated. Some authors have proposed several hypotheses, including surgically induced wound gape [28], removal of scleral tissue [11], the role of sutures and suture lysis [12], the influence of postoperative intraocular pressure (IOP) [31], and the pressure exerted by the eyelid and bleb on the cornea surface [32]. Moreover, there is insufficient information on the astigmatic effects of other minimally invasive glaucoma surgeries. To the best of our knowledge, this study is first to investigate both keratometry and ACD changes following XEN surgery.

Our findings distinctly highlight that XEN implantation does not lead to changes in keratometry or BCVA outcomes. This procedure involves the use of mitomycin C and the development of a bleb, while not necessitating sutures or structural alterations to the eye for successful outcomes. This suggests that induced astigmatism following glaucoma procedures could likely be attributable to structural changes in the eye and the use of sutures, with mitomycin C and bleb formation playing negligible roles. In light of these findings, the lack of significant induced astigmatism supports the feasibility of combining cataract and XEN implantation surgeries without the risk of unaccurate refractive outcomes.

Nonetheless, our study has several limitations. First, a small sample size was evaluated; this study helps to plan prospective studies with a sufficient sample size to detect possible differences between trabeculectomy and XEN or other device implantation. Moreover, the follow-up time was only 3 months and further studies investigating long-term outcomes are required.

## 5. Conclusions

The present study revealed that the XEN implantation did not result in a decline in the visual acuity or changes in keratometry or ACD. Patients undergoing XEN implantation could hence be reassured about this aspect. Moreover, this research provides evidence that this surgery is both effective and safe for patients with open-angle glaucoma.

## Figures and Tables

**Table 1:** Demographic data. The statistical analysis indicated no significant differences between the study and control groups (*p* > 0.05).

	Study Group (*n* = 20) *n* (%)	Control Group (*n* = 20) *n* (%)	*p*-Value
Gender			
Male	12 (60%)		
Female	8 (40%)		
Mean Age (SD)	64.3 (11.25)		
Eye			0.75
Right	11 (55%)	9 (45%)	
Left	9 (45%)	11 (55%)	
Previous Ocular Surgery			0.75
Phaco + IOL	7 (35%)	8 (40%)	
PPV	1 (5%)	0 (0%)	
Lens Status			1
Phakic	13 (65%)	12 (60%)	
Pseudophakic	7 (35%)	8 (40%)	
Surgical Procedure			
XEN standalone	16 (80%)		
XEN + Phaco	4 (20%)		

**Table 2:** Baseline and follow-up results for the mean ACD, ACV, astigmatism, and steep during the 3 months of follow up.

		Study Group (*n* = 20)	Comparison to Baseline *p*-Value (Pairwise Signed Rank Test)	Control Group (*n* = 20)	Comparison to Baseline *p*-Value (Pairwise Signed Rank Test)	Intergroup Comparison *p*-Value
AC depth (mm)	Baseline	3.37 ± 0.59		3.44 ± 0.48		0.67
	POD 1	3.27 ± 0.55	0.30	3.45 ± 0.46	0.83	0.28
	POD 7	3.25 ± 0.54	0.35	3.38 ± 0.44	0.28	0.41
	POD 14	3.30 ± 0.56	0.40	3.41 ± 0.43	0.22	0.47
	POM 1	3.30 ± 0.59	0.22	3.41 ± 0.46	0.07	0.49
	POM 2	3.23 ± 0.51	0.07	3.29 ± 0.39	0.12	0.66
	POM 3	3.32 ± 0.51	0.43	3.38 ± 0.43	0.11	0.69
AC volume (mm^3^)	Baseline	169.30 ± 39.36		170.55 ± 34.81		0.91
	POD 1	161.90 ± 31.57	0.51	171.15 ± 33.40	0.46	0.37
	POD 7	160.60 ± 33.95	0.33	166.05 ± 28.33	0.34	0.58
	POD 14	164.40 ± 39.75	0.43	171.30 ± 33.89	0.51	0.55
	POM 1	165.85 ± 41.31	0.49	171.30 ± 36.51	0.45	0.66
	POM 2	168.05 ± 40.43	0.83	161.95 ± 29.82	0.45	0.64
	POM 3	170.90 ± 35.34	1	171.10 ± 26.52	0.69	0.72
Astigmatism	Baseline	0.94 ± 0.64		0.80 ± 0.52		0.62
	POD 1	0.91 ± 0.44	0.32	0.77 ± 0.46	0.93	0.34
	POD 7	0.89 ± 0.40	0.96	0.73 ± 0.44	0.17	0.23
	POD 14	0.91 ± 0.41	0.53	0.72 ± 0.48	0.09	0.19
	POM 1	0.93 ± 0.41	0.88	0.75 ± 0.52	0.57	0.12
	POM 2	0.87 ± 0.42	0.93	0.75 ± 0.50	0.51	0.24
	POM 3	0.87 ± 0.39	1	0.80 ± 0.56	0.97	0.35
Steep	Baseline	99.3 ± 43.7		97.1 ± 45.5		0.87
	POD 1	91.8 ± 37.9	0.98	104.3 ± 40.3	0.30	0.31
	POD 7	89.3 ± 37.3	0.61	110.7 ± 44.8	0.13	0.10
	POD 14	91.3 ± 37.3	0.69	102.1 ± 43.4	0.08	0.31
	POM 1	88.7 ± 31.4	0.53	107.5 ± 41.6	0.07	0.08
	POM 2	89.5 ± 33.6	0.48	101.2 ± 42.9	0.48	0.34
	POM 3	84.8 ± 31.9	0.77	98.0 ± 40.1	0.92	0.18

## Data Availability

The original contributions presented in the study are included in the article, further inquiries can be directed to the corresponding author.
